# Food Recipe Ingredient Substitution Ontology Design Pattern

**DOI:** 10.3390/s22031095

**Published:** 2022-01-31

**Authors:** Agnieszka Ławrynowicz, Anna Wróblewska, Weronika T. Adrian, Bartosz Kulczyński, Anna Gramza-Michałowska

**Affiliations:** 1 Center for Artificial Intelligence and Machine Learning (CAMIL), Faculty of Computing and Telecommunications, Poznan University of Technology, 60-965 Poznań, Poland; 2 Faculty of Mathematics and Information Science, Warsaw University of Technology, 00-662 Warsaw, Poland; anna.wroblewska1@pw.edu.pl; 3 Applied Computer Science Department, Faculty of Electrical Engineering, Automatics, Computer Science and Biomedical Engineering, AGH University of Science and Technology, al. A. Mickiewicza 30, 30-059 Krakow, Poland; wta@agh.edu.pl; 4 Department of Gastronomy Science and Functional Foods, Faculty of Food Science and Nutrition, Poznań University of Life Sciences, 60-637 Poznań, Poland; bartosz.kulczynski@up.poznan.pl (B.K.); anna.gramza@up.poznan.pl (A.G.-M.)

**Keywords:** food computing, ontology design pattern, substitute model, artificial intelligence

## Abstract

This paper describes a notion of substitutions in food recipes and their ontology design pattern. We build upon state-of-the-art models for food and process. We also present scenarios and examples for the design pattern. Finally, the pattern is mapped to available and relevant domain ontologies and made publicly available at the ontologydesignpatterns.org portal.

## 1. Introduction

People may want to substitute ingredients in recipes for several reasons, including constraints and goals. Constraints can be eliminating food people are allergic to or temporarily lacking in their household. Goals can be increasing the intake of a particular nutrient or making the dish less dense.

Another vital aspect of substitutions in recipes are the technological properties of ingredients. For example, a sponge cake recipe contains eggs and sugar. However, people with diabetes should limit their intake of sugar. Therefore, for the sake of the diet, sugar should be replaced by another food product to keep the same sweetness. In preparing this recipe, the technological properties of the substitute must also be taken into consideration, as the cake must rise.

The computational support for handling tasks, such as recommending ingredient substitutes, falls into a relatively new area of *food computing* [1] with a broad spectrum of applications, such as food recognition [2], detecting food intake with the use of sensors [3], and *computational diet management* [4]. Many recent approaches in food computing and diet management use artificial intelligence (AI), both statistical methods (based on various forms of machine learning) and symbolic ones (based, for instance, on the usage of knowledge graphs [5]).

However, the current works on using AI for food recipe ingredient substitution are, so far, very scarce. Pan et al. [6] examined how to use natural language processing techniques, such as word embeddings, to find alternative components in a data-driven, similarity-based manner. Several systems, on the other hand, incorporated explicit semantic information about ingredients and explicit rules. Gaillard et al. [7,8] developed TAAABLE to conduct recipe modifications based on explicit rules and knowledge about ingredient subclass taxonomy using techniques such as Formal Concept Analysis. Skjold et al. [9] proposed Intellimeal, a case-based reasoning system for recommending recipes, with the main focus of customizing recipes to a given user query re-using the domain knowledge with adaptation rules. Recipe adaptations were performed by searching for recipes that most closely matched a given user query and then performing modifications based on ingredient taxonomy similarity and substitution rules. Shirai et al. [10] coupled explicit semantic information with implicit information (embeddings) to create the DIISH heuristic for ingredient substitutability, which provides substitutes depending on user context linked to health.

However, *none of these works defines an explicit, semantic model of an ingredient substitution phenomenon, capturing it as a design pattern*. Such a pattern could be a prerequisite to systematically capture in AI-based systems all the aspects that need to be taken into account when proposing an ingredient substitution, as well as to integrate the relevant concepts from available data and knowledge sources (such as ontologies and knowledge graphs).

### 1.1. Food Substitutes and Analogs

Food and nutrition sciences distinguish *substitutes* and *analogs*. A *substitute* is a product subject to substitution, i.e., the replacement of one or more components, the technological process of which remains unchanged, resulting in a product similar to the original. In turn, an *analog* is an imitation, which is a product that is substituted in the sense that one or even all of the ingredients are replaced, and also the technological process is changed, the result being a product that imitates the original product in terms of smell, texture, and/or taste.

The compositional characteristics of the product indicate the specific components of the recipe in the form of raw materials, additives, and cooking techniques used to direct the specific sensory, as well as functional characteristics of the product. These raw materials may be substituted with other raw materials or products, the use of which will not significantly change the characteristics of the final culinary product but may change its nutritional value or desired dietary characteristics, with the lowest possible change in sensory quality.

Each recipe may have obligatory ingredients, which will determine the use of the recipe, but this problem must be considered from various levels. Assuming that the beef stew contains meat as an obligatory ingredient; then its replacement with other meat, such as goat or sheep meat, may change the sensory characteristics and nutritional value of the final product. In this particular case, we are replacing the meat of one animal with another; definitely, a different situation occurs when we replace meat with a meat analog, e.g., soy texturate. The use of soy as a substitute for meat causes that the obligatory ingredient may no longer be an obligatory one and becomes an optional one in a new recipe, which, however, with the use of the remaining ingredients of the recipe, allows to obtain the final product similar in its sensory and nutritional features to the initial product.

The above possibilities are one of the important elements of creating innovations in recipes; however, it is very difficult to capture as a universal application. It is important that the potential user is given several options to choose from, with each choice being justified by the reasonableness of the choice within the framework of the sensory attributes, nutritional value, and technological feasibility of the product or the combination of raw materials included and described previously. The decision to use a given substitute is up to the user of an application, his/her preferences (taste, aroma, texture, etc.) and needs (vegetarians, obese, diabetics, etc.). If, on the other hand, one considers the possibility of modeling cultural factors influencing the choice of a substitute, the focus should be on the available choice options, and regional traditions, religious backgrounds, and other culturally empowered decisions that belong to the user of the application. Again, the characteristics of the ingredient being substituted and the substitute itself must be considered when describing the characteristics of the substitute. The basis for proper and effective ingredient substitution in a recipe is prior knowledge of the specific technological and dietary characteristics that determine substitution in a particular recipe and ingredient mixture to achieve the desired effect.

One must also consider situations where there are variations of recipes with non-typical ingredients, for example, when a dish of meat is cooked as a vegan one by using mushrooms instead of meat; however, such substitutions may apply only to one type of dish with meat as a main ingredient and to no other. Based on the knowledge of food technology and dietetics, it is possible to suggest in advance the specific substitutes for the product ingredients appearing in the recipes. The selection of substitutes would be dictated by similar nutritional value and/or similar sensory characteristics and the technological function the product performs in the dish. For example, beef can be replaced with poultry meat, pork, venison, lamb, etc. As mentioned, using a strictly defined group of substitutes, the consumer will decide for himself/herself which product he/she would like to replace the given ingredient with. At the same time, each permitted form of substitution will be accompanied by a comment on how the final nutritional value of the finished dish will change (e.g., the modified dish will have lower calories than its original counterpart). Therefore, a proposal to substitute an ingredient for another must involve a finite set and be limited to ingredients that condition the production of a final product with similar characteristics to the initial product of which the ingredients are being substituted. An important feature, then, is simple food modeling, in which a resource constraint on the characteristics of the substitute will result in a facilitated user decision about the potential substitute.

### 1.2. Objectives and Contributions

In this work, we undertake a foundational analysis of ingredient substitution and investigate how to capture and model it explicitly. We are, in particular, interested in the following main research question:

- How to model substitutes for ingredients in food recipes?

This research question can be broken down into more specific questions, such as:

- What related concepts should be taken into account to define the substitution’s context (e.g., conditions, goals)?
- How to link the proposed model to existing food models and recommended design patterns?

As a result of this analysis, we propose a data model and structure recipe ingredient substitution with an ontology design pattern. The objectives for this data model are as follows:

- to document recommended design patterns,
- to ensure existing food ontology network compatibility,
- to ensure OBO relation ontology compatibility.

Thus, our contributions are: ontology design patter for substitutes in food recipes, usage scenarios, and an example.

## 2. Materials and Methods

Food recipes have a format that includes: a title, a list of ingredients, and step-by-step instructions. The title should appropriately describe a recipe and explain its content. The ingredients list must include the quantity, unit name, and food item name. The quantity of all ingredients must correspond to the number of servings specified in the recipe. The unit’s name must be related to the quantity and suitable for the ingredient form (liquid, dry countable, dry uncountable, with or without unit). The instructions section must correctly present the order of steps. In addition, the processing of each ingredient must be considered in the subsequent recipe steps, which must reflect the status of the ingredient after the processing.

In order to model the process of substituting ingredients in recipes, we consider two main categories of materials: (1) available food ontologies and knowledge graphs and (2) recipe datasets. The former provides us with modeling guidelines and existing modeling patterns at the schema level. The latter provides us with data.

Considering the methods, we will use the methodology of ontology design patterns.

### 2.1. Food Ontologies and Knowledge Graphs

OBO (Open Biological and Biomedical Ontology) Foundry [11] is a consortium of interoparable life science-oriented ontologies. Among others, it supports the development of an ecosystem of food-related ontologies, including ontologies dedicated to food production, agriculture, dietary nutrition, food interaction with drugs, manufacturing processes etc. This ecosystem shares common relations and concepts, focusing on different aspects of provenance, preparation, and consuming food [12].

A central food ontology (called ’The Farm to Fork Food Ontology’) and a main reference for food products is FoodOn [13]. (See https://foodon.org/ (accessed on 27 January 2021)). It provides semantics for a range of food-related topics, including food production, culinary, nutritional and chemical ingredients, and processes. The ontology re-uses terms from other OBO Foundry ontologies on agriculture, plant and animal anatomy, or nutritional components, and on the other hand, its terms are re-used in ontologies on nutritional studies (ONS) [14] and epidemiology (ONE) [15], Food Interactions with drugs (FIDEO) etc. FoodOn was based largely on a descriptive food indexing system (thesaurus) called LanguaL (See https://www.langual.org/ (accessed on 27 January 2021)). Different sub-domains can be explored via FoodOn facets.

**Figure 1:**
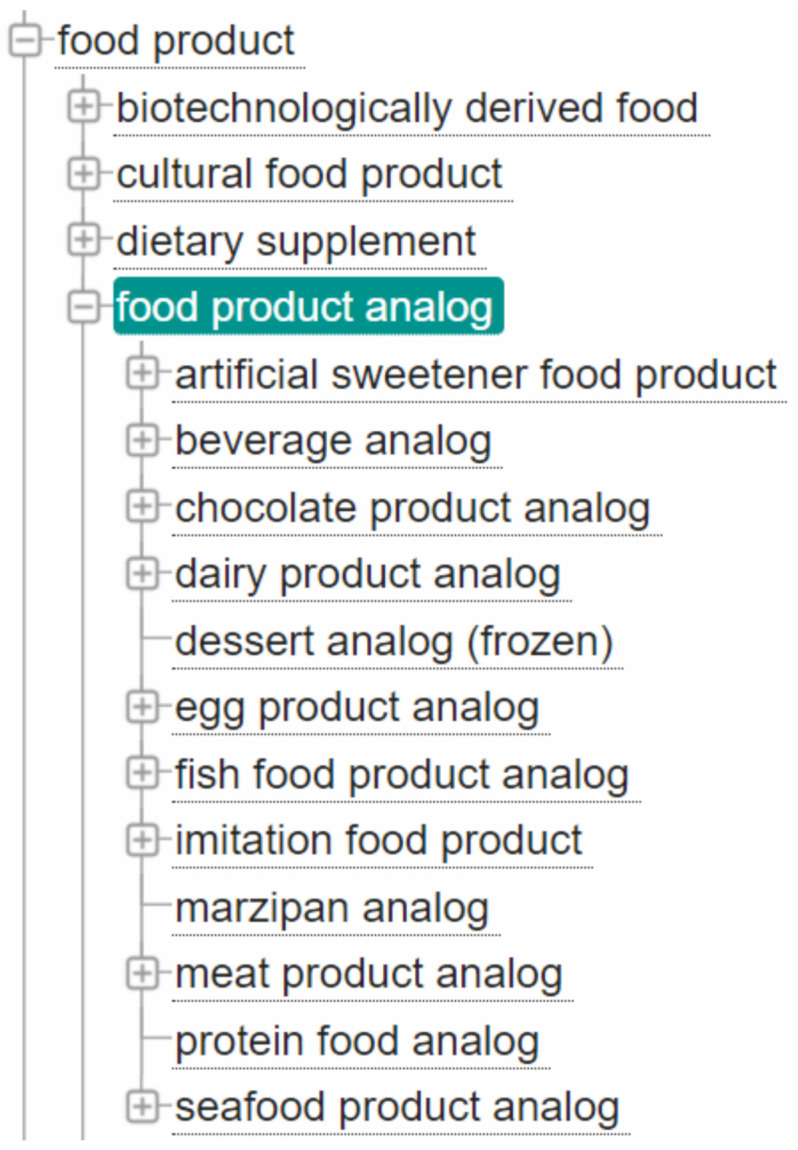
An excerpt from the FoodOn ontology with the subhierarchy of food analogs classes.

In order to connect ingredients to substitutes for use in allergy and other dietary constraints analyses and applications, FoodOn provides a ’food substance analog’ relation that can connect any two *food source items* or *products*, inviting substitution. The relation is said to be symmetric, i.e., it is not precise which of the item is a substitute of the other. However, the relation itself is not well described; there are no formal statements about its properties or even the domain and range of it. Furthermore, nothing is said about the quality of the substitution or appropriate ratio. Finally, as the rationale for including substitute concepts stems from the nutritional analysis, the terms are linked to the Disease Ontology (DO (See https://disease-ontology.org/ (accessed on 27 January 2021))), but nothing more (e.g., the substitution is not analyzed within a recipe process context). To complete the picture, in FoodOn, products can be explicitly defined as ’food product analog’ (subclass of ’food product’), a class that has multiple subclasses, including ’artificial sweetener food product’, ’chocolate product analog’, ’meat product analog’, etc. (see Figure 1).

The problem of substitution has been analyzed in more detail by Shirai et al. [10]. The authors have defined the objective of finding and ranking substitutes in the contexts of two motivating cases: (1) personal dietary restrictions satisfaction and (2) modification of the nutritional contents of meals. The main data source for the method proposed in this work is FoodKG [16]—a knowledge graph that covers recipes and ingredient information. To achieve the goal of proposing a good ingredient substitution, the possible food items (e.g., food items appearing in similar recipes) from the FoodKG have been linked with:

- concepts from FoodOn for food categorization, and
- semantic descriptions from the USDA (See https://catalog.data.gov/dataset/food-and-nutrient-database-for-dietary-studies-fndds (accessed on 15 December 2021)) for their nutritional information.

As a result, the authors propose a Diet-Improvement Ingredient Substitutability Heuristic (DIISH) that combines nutrition calculation based on explicit semantic information and latent semantic analysis. This method, although using a multidimensional analysis, is not based on any explicit formal conceptualization of the ingredient substitution. Somehow, surprisingly, in nutritional ontologies ONE and ONS that contain concepts about dietary regimes etc., no concepts or relations are defined for substitutions (see Table 1).

### 2.2. Recipe Datasets

Available recipe datasets are mainly focused on information retrieval, (image-text) recipe generation, or overall information about cooking and ingredients. In paper [1], Min et al. survey existing benchmark food datasets. The most extensive dataset is Recipe1M+ dataset [17] or RecipeNLG [18].

Another dataset worth mentioning is FoodBase [19], an annotated corpus of food entities, in which food products are indicated and linked to FoodOn ontology, constructed using recipes from Allrecipes, the largest food-focused network.

### 2.3. Ontology Design Patterns

Patterns can be characterized broadly as unique and repeated invariants across observable data, objects, and processes that are either made or arise spontaneously. Design patterns arose in computer science and were first used in software engineering, then also in data modeling and ontology engineering, where they are called Ontology Design Patterns (ODPs) [20].

Content patterns are the most prevalent sort of pattern, which we will refer to when using the ODP acronym. A content pattern is essentially equivalent to a software design pattern, but it also contains a reference basic implementation that is ready for rapid customization. These are often modeled for regularly recurring features of more complicated ontologies and serve as building blocks. Gangemi [21] and Blomqvist and Sandkuhl [22] proposed Content Ontology Design Patterns to facilitate ontology construction. They are meant to help non-expert users by bundling best practices into reusable blocks of functionality, which such users may tweak and specialize in their ontology development projects.

## 3. Design Considerations

We considered several issues while making design choices for modeling the ingredient substitutes. The discussion of these issues is structured into the following subsections.

### 3.1. Aspects of a Dish

**Figure 2:**
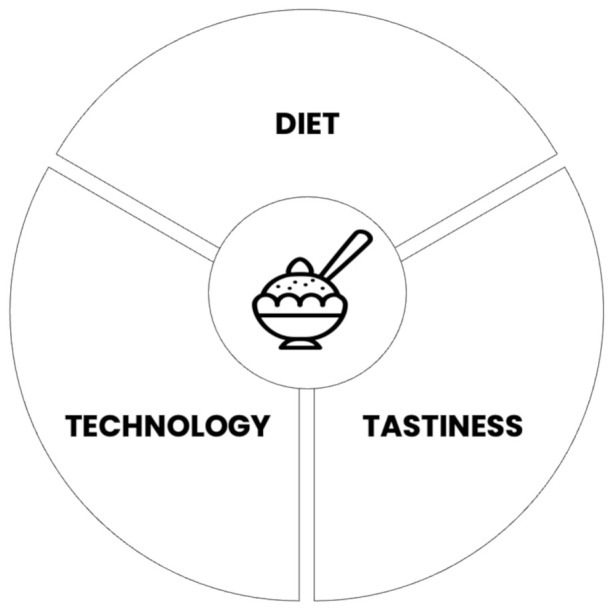
Aspects of a dish to consider while replacing ingredients.

When preparing a dish based on a given recipe, a cook can make a substitution because he/she has no such particular ingredient at hand or has dietary constraints due to health issues or other preferences. Table 2 shows a few scenarios in which a cook must do a substitution for different reasons (see Figure 2). The substitution should also be performed with proper proportions and cooking conditions. Moreover, the dietary effect, such as nutrition values or technological effect, are different.

### 3.2. Example

Let us further consider a sample recipe (Table 3) and sample recipe substitution (Table 4).

Table 3 shows an example recipe for pancakes. In this recipe, products can be substituted for reasons of taste, technology, or diet. For example, sugar can be replaced by erythritol for diabetics (for dietary reasons). We can take care of the taste and replace the frying oil with butter. However, with the ingredient—frying oil—we also have a restriction—the frying oil must not be cold-pressed oil. For gluten-free people, we can replace the all-purpose flour with gluten-free flour, then the technology will also change a bit. For those who are allergic to milk, we can change the milk for plant milk. If we replace the melted butter with regular butter, we need to add a pre-processing step—melting the butter—to the recipe process itself (see Table 4).

The proposed substitution has two parts: (1) replacing ingredients (e.g., **‘butter, melted’** into **‘regular butter’**, and (2) adjusting the instructions to encompass ingredient change (e.g., adding a pre-processing step consisting of melting butter previously not melted). These need to be reflected when modeling a pattern.

### 3.3. Modeling Processes in OBO

To cover the process modeling space, OBO employs a number of ontologies, the most important of which are as follows:

- Ontology for Biomedical Investigations (OBI) [23] developed the concept of ‘planned process’ [OBI_0000011].
- Information Artifact Ontology (IAO), which grew out of OBI [24], addresses data items throughout the input/output operations.
- Relations Ontology (RO) [25] expresses the majority of the relations that accompany a process.

The recently proposed FoodOn recipe model [26] incorporates numerous additional components, such as ‘ingredient set’, ‘device set’, and ‘instruction set’. These sets are planned to be subclasses of IAO **‘data set’** [IAO_0000100], which is a data item that is an aggregate of other data items that have something in common.

An important modeling distinction is made between processes specification and their actual execution.

## 4. Recipe Ingredient Substitution Pattern

### 4.1. Intent

Our primary intent is to model substitutes for ingredients in food recipes. Essential aspects of modeling substitutes are their quantity in a recipe, the constraints and the qualitative and quantitative conditions for the substitutions, and other nutrition values, e.g., containing a number of calories or technological effects, and substitution objectives.

The pattern should allow the representation of different types of food substitutes in recipes, recipe process, and overall notion of food substitution.

### 4.2. Competency Questions

The list of competency questions for the pattern is as follows:

1. What ingredient is being replaced with another ingredient or a set of ingredients?
2. What are the dietary features of the ingredient?
3. What are the food technology features of the ingredient?
4. What are the nutrition values the ingredient has?
5. What is the quantity of the ingredient being processed in the recipe? What is the unit of the quantity?
6. What is the objective of ingredient substitution?
7. What conditions (dietary, technological) are specified for selecting the target ingredient?
8. How is the ingredient processed in the given recipe?
9. What is the ratio for substitution of one ingredient into another?
10. What ingredient substitutions are possible in the recipe?
11. What ingredient substitutions are available in the recipe given dietary constraints?
12. What are available substitutes for a given food item? What are substitutes for the ingredient in the recipe?

### 4.3. Graphical Representation

**Figure 3:**
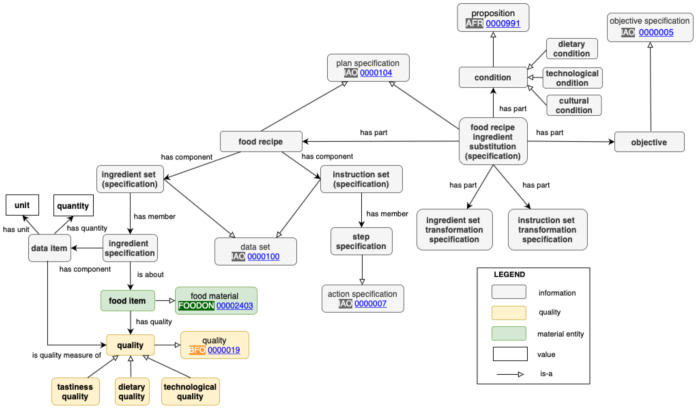
Graphical representation of the pattern and mapping to relevant terms from existing ontologies.

The Food Recipe Ingredient Substitution Ontology Design Pattern is depicted in Figure 3. The classes belonging to the pattern are marked by bold font, while the others present the alignment to the classes from relevant, existing ontologies.

### 4.4. Basic Conceptual Entities

A **‘food recipe’** provides a procedure on how to prepare a dish. In this way, it is not a process per se (spanning a time period) but rather a specification of a process to be performed to prepare a dish. Therefore, a major modeling decision regarding the pattern is to model ingredient substitution in recipes on the level of *specification* rather than processes. A **‘food recipe’** has two major components important from the point of view of modeling substitution: the ingredient set and the instruction set.

An *ingredient* is a complex entity since it usually consists of not only food items (materials), but also a specification of a quality measure of unit and quantity. When ontologically modeling an ingredient on the level of specification, it becomes an **‘ingredient specification’**. It may specify an ingredient that is a simple one or a complex one, the latter expressed by a logical formula. A complex ingredient may be, for instance, a disjunction of ingredients (’goat or sheep meat or both’) or a conjunction, such as a conjunction of aromatics, for instance, ’dill and fennel’, ’green and dry onions’, or combinations of spices. In practical settings, some ingredients in a recipe may be *optional*, although this concept is not explicitly defined in the Food Process Ontology [26] we refer to. However, in the case one would like to substitute an ingredient that is optional, an ’empty’ substitution could be proposed that would mean that the ingredient should simply be omitted in the final recipe. On the other hand, one way of modeling obligatory ingredients may be by using the property included in FoodOn ’has defining ingredient’, which is meant to represent ingredients that are obligatory in the food product, such as a carrot in a carrot cake. This way of modeling can be adapted to the level of specification and reflected by introducing the ’has obligatory member’ property. On the level of ingredient specification, an obligatory ingredient may also be also modeled by assigning an attribute (i.e., binary property) ’obligatory’ to the ingredient specification.

Ingredient specification refers to a material **‘food item’**, representing physical food. Furthermore, a physical **‘food item’** may have various characteristics (qualities) that are represented in the pattern by the class **‘quality’**. These may be characteristics such as: color, shape, being whole or divided, physical state, or degree of maturity, including such technological characteristics as ‘for frying’, ‘for baking’, etc. **‘Quality’** may also include features such as fat content in meat (below or above 10%) or sugar content and dietary characteristics (e.g., gluten-free, vegetarian, etc.). Moreover, the sensory characteristics of the product, as well as of the ingredient substitute, linked to tastiness (such as taste, aroma, mouthfeel [27]), can be defined as characteristic of the raw material itself, as well as of its characteristics after processing. Sensory acceptability of a product is specific and individual, depending not only on consumer preferences but also on many other factors, e.g., culinary habits, physiological or psychological state. The determination of sensory characteristics is very difficult to present at a high level of generalization because such an evaluation can be conducted with different methods and with a different group of consumers, including a panel of trained evaluators of these characteristics, as well as the average consumer evaluating a product based on his/her own sensory preferences. Accordingly, when considering that users are selecting ingredients to substitute, the user may be provided with several pieces of information previously included in a product sheet that will help the user optimize the selection of a particular substitute within the desired sensory and functional characteristics. To this end, we have included in the model subclasses of **‘quality’** extending the model with tastiness and technological and dietary qualities of ingredients. Three aspects presented in Figure 2 are reflected as sub-classes of **‘quality’**: **‘technological quality’**, **‘dietary quality’**, and **‘tastiness quality’**. **‘Unit’** and **‘quantity’** further characterize a particular ingredient in the recipe. They are linked to **‘data item’**, a term appearing in IAO and representing information collected for some purpose, such as measurement recording categorical, numerical or unit values, and intended to be a truthful statement about something. The unit may represent a dimension of what is measured. Then if the unit is, for instance, ‘cup’, the quality being measured is volume. Following recent modeling proposals [26], we model a set of ingredients in the recipe as **‘ingredient set (specification)’**, while a particular ingredient specification is its member.

**‘Instruction set (specification)’** has members **‘step specification’**.

Based on the analysis of our motivating scenarios, we have concluded that modeling ingredient *substitutes* have two major settings: (a) without context, i.e., as general substitutes and (b) taking into account the context of a specific recipe and its food technological aspects and other factors, such as dietary constraints, goals, and technological conditions. In the latter case, the relation expressing **substitution** becomes *n*-ary, as it not only represents two ingredients, one to be replaced with another one but also conditions and (planned) effects of such a substitution. To model an *n*-ary relation using ontology modeling language OWL, it needs to be reified to a class [28,29]. When it comes to applications such as recommendations of ingredient substitutes in a recipe, the actual substitution is *suggested* to the user; therefore, it is still on the level of the specification: **‘food recipe ingredient substitution (specification)’**.

**‘Food recipe ingredient substitution (specification)’** has parts reflecting conditions and planned effects (objectives) of substitution.

**‘Condition’** represents various constraints to be met for the target ingredient to be recommended. These may be technological and dietary constraints reflected by classes **‘technological condition’** and **‘dietary condition’**, respectively, or even **‘cultural condition’**. Considering the former ones, when substituting ingredients that are important for the organoleptic characteristics of the final dish, the technological functions of the substituted ingredient should be taken into account, e.g., sugar guarantees; appropriate color (browning of bread crust), texture, shelf life, etc. Another example may be to consider rapeseed oil as a substitute for olive oil, but the condition for this substitution is that the extra-virgin olive oil cannot be used for heat treatment (e.g., frying, baking) due to its technological suitability. Considering the latter ones dealing with dietary constraints, then, for example, ground beef will not be offered to a vegetarian as a substitute for chickpeas.

**‘Objective’** represents various goals and planned effects to achieve by ingredient substitution. These may be most important objectives regarding dietary and health goals, such as ‘increased fiber’ in the resulting dish or ‘excluded sugar’ (due to health requirements related to type 2 diabetes).

Major further parts of the proposed substitution model, replacing ingredients and adjusting the instructions to encompass ingredient change (see Table 4), are further reflected in the pattern by the classes: **‘ingredient set transformation (specification)’** and **‘instruction set transformation (specification)’**.

### 4.5. Alignment of the Pattern with Existing Ontologies

When developing the pattern, we have paid special attention to aligning the proposed modeling with the classes from existing, relevant ontologies and relations from OBO relation ontology. Below we describe the mapping to relevant classes (depicted in Figure 3):

**‘food recipe’**: ONS (Ontology of Nutritional Studies) re-uses the term **‘recipe’** [SIO_001042] from the Semanticscience Integrated Ontology (SIO) [30] for biomedical research and knowledge discovery and makes it a subclass of **‘plan specification’**[IAO_0000104], the term from IAO.

**‘food item’**: Food items may be mapped to **‘food material’** [FOODON_00002403], which represents substances that can be consumed by an organism to satisfy nutritional or other health needs, or to provide a social or organoleptic food experience.

**‘quality’**: To define an object’s observable property, there are used terms, such as ‘characteristic’, ‘feature’, ‘quality’, ‘attribute’, or ‘phenotype’. In the biological sciences, BFO utilizes ‘quality’ rather than ‘characteristic’ for an observable object trait within OBO; we used that term for the mapping. When it comes to food science in particular, FoodOn also uses the term ‘quality’ from PATO ontology.

**‘step specification’**: Particular instructions on the level of the specification may be mapped to **‘action specification’** [IAO_0000019].

**‘condition’** is modeled by AFO, a set of ontologies that offers a semantic model for representing laboratory analytical processes (Equipment, Material, Process, and Results), as an **‘information content entity’** that ‘*is about the portion of reality under which something occurs or is valid*’. The AFO suite is aligned with the Basic Formal Ontology (BFO) and models **‘condition’** as a subclass of **‘proposition’**, defined as an **‘information content entity’** that is ‘*a statement or assertion that has a truth value*’. It is well-aligned with our envisaged use of this term. SIO also models **‘condition’**, specifying it with further axiomatization.

We mapped **‘objective’** to **‘objective specification’**[IAO_0000005].

Finally, **food recipe ingredient substitution (specification)** is also per itself a **‘plan specification’** [IAO_0000005], as it specifies both the plans to transform the ingredient set and instruction set.

## 5. Conclusions

In this paper, we have proposed an ontology design pattern for modeling ingredient substitution in food recipes.

Our analysis of the existing work revealed that although several works propose AI techniques for recommending substitutes, there has previously been no fundamental, ontological analysis of the substitution phenomenon. Existing knowledge resources though sometimes model ’substitute’ relation, do not provide its conceptual definition and treat this relation as a binary relation between two generic substitutes.

After analyzing several motivating scenarios and examples and related process models, we have concluded that ’substitution’ should be modeled as a class, after reifying it as an *n*-ary relation that tackles contextual features of substitution, such as not only ingredients to replace with each other, but also conditions and objectives of such a replacement.

In proposing the pattern, we have tried to build upon state-of-the-art models for food and process. We also mapped the pattern to available and relevant domain ontologies and made it publicly available at the ontologydesignpatterns.org portal. We hope that the result of this modeling effort will prove helpful as a prerequisite for further ontological modeling of food substitution aspects and for developing data and schema models for AI applications for capturing and recommending ingredient substitutes.

## Figures and Tables

**Table 1:** Conceptualization of substitutions in existing ontologies and knowledge graphs.

Ontology	Substitution	Context	Limitations
FoodOn [13]	a (symmetric) relation: ’has food substance analog’	dietary and allergen analysis	no links with food preparation process, recipes
	subclasses of class: ’food product analog’		
FoodKG [16]	heuristics based on explicit semantics and embeddings	dietary restrictions nutritional change	no ontological conceptualization
ONE [15]	no term(s) for substitution		
ONS [14]	no term(s) for substitution		

**Table 2:** Motivating scenarios.

**Scenario 1:** Substitution due to lack of product.
**Objective:** In this scenario, the substitute must replicate nutritional value, i.e., also be a good source of fiber.
**Description:** Persona X decided to prepare risotto. However, when preparing the dish, she found that she had run out of brown rice. The recipe she used did not provide any information on the ingredient(s) with which the rice could be replaced. Furthermore, Persona X has decided to eat healthily and wonders which possible rice substitute would meet her expectations, i.e., would not only be technologically suitable but above all would be a good source of dietary fiber.
**Scenario 2:** Substitution due to the need to exclude a particular product for health reasons.
**Objective:** In this scenario, the substitute must meet health requirements.
**Description:** Persona Y decided to make a dairy dessert with fruit for a birthday party. However, it turned out that one of the guests had a diagnosed allergy to cow’s milk protein and nuts. In order not to completely abandon the idea of preparing a sweet snack, Persona Y decided to replace the milk with another ingredient. Unfortunately, she has no idea which product would make a good substitute for milk. Her husband has given her the idea that it could be an almond drink. However, she is not convinced that it would be a suitable replacement as the guest has a diagnosed nut allergy.
**Scenario 3:** Substitution due to the need to exclude a specific product for health reasons.
**Objective:** In this scenario, the substitute must meet the technological requirements, i.e., it must give the same sweetness to the dish as sugar.
**Description:** Persona Z is in the process of preparing baked cookies for a family gathering. However, she finds out that one of the participants will be her grandmother—type 2 diabetes. Therefore, she decided not to use added sugar in the baked goods. Unfortunately, the younger guests would not appreciate cookies without sugar’s sweet taste. Therefore, Persona Z wondered what she could do to replace the sugar in the cookies to keep them sweet. Admittedly, her sister has suggested that erythritol is a popular sugar substitute in recent times. However, two doubts remained to be resolved: (1) will erythritol not lose its sweetness during baking, (2) in what ratio to replace sugar with erythritol to get a similar sweetness?

**Table 3:** Example: A recipe for pancakes.

**Ingredients for 8 serving**
all-purpose flour—1½ cups
salt—pinch or more to taste
white sugar—1 tablespoon
milk—1¼ cups
egg—1
butter, melted—2 tablespoons
oil for frying
**Recipe process**
Step 1. Mix flour, salt, and sugar in a bowl. Add milk, egg, and melted butter.
Step 2. Blend until smooth.
Step 3. Heat a frying pan with light oil.
Step 4. Pour the batter into the pan, using about 1/3 cup for each pancake.
Step 5. Brown on both sides.

**Table 4:** Example substitution for a recipe for pancakes and the changed recipe. (Source recipe in Table 3).

**Substitution**
butter, melted—>regular butter
Pre-processing step. Melt butter.
**Ingredients**
all-purpose flour—1½ cups
salt—pinch or more to taste
white sugar—1 tablespoon
milk—1¼ cups
egg—1
**butter—2½ tablespoons**
oil for frying
**Recipe process**
**Step 0. Melt butter.**
Step 1. Mix flour, salt, and sugar in a bowl. Add milk, egg, and melted butter.
Step 2. Blend until smooth.
Step 3. Heat a frying pan with light oil.
Step 4. Pour the batter into the pan, using about 1/3 cup for each pancake.
Step 5. Brown on both sides.

## Data Availability

The pattern has been made publicly available at the repository OntologyDesignPatterns.org (accessed on 26 January 2022), a Semantic Web portal dedicated to ontology design patterns at http://ontologydesignpatterns.org/wiki/Submissions:Food_Recipe_Ingredient_Substitution_Ontology_Design_Pattern (accessed on 26 January 2022).

## Abbreviations

The following abbreviations are used in this manuscript:

OBO	Open Biological and Biomedical Ontology
FoodOn	The Farm to Fork Food Ontology
ONS	ontologies on nutritional studies
FIDEO	Food Interactions with drugs
DO	Disease Ontology
FoodKG	a knowledge graph that covers recipes and ingredient information
USDA	the U.S. Department of Agriculture
FNDDS	food and nutrient database for dietary studies
DIISH	Diet-Improvement Ingredient Substitutability Heuristic
AI	Artificial Intelligence
TAAABLE	Taaable system addresses the mixology and the sandwich challenges [7]
LanguaL	food thesaurus
Recipe1M+	dataset
RecipeNLG	dataset
ODP	Ontology Design Patter
RO	Relations Ontology
IAO	Information Artifact Ontology
OBI	Ontology for Biomedical Investigations
SIO	Semanticscience Integrated Ontology
BFO	Basic Formal Ontology
PATO	Phenotype And Trait Ontology
AFO	ontology that interoperates with the Basic Formal Ontology (BFO) and the Relation Ontology (RO)
